# Predictive factors in difficult postoperative airway management of severe odontogenic deep neck infection

**DOI:** 10.1007/s10266-024-01041-w

**Published:** 2024-12-14

**Authors:** Eiji Iwata, Go Inokuchi, Masakazu Kawakami, Taiki Matsui, Junya Kusumoto, Akira Tachibana, Masaya Akashi

**Affiliations:** 1 Department of Oral and Maxillofacial Surgery, Kobe University Graduate School of Medicine, Kobe, Japan; 2 Department of Oral and Maxillofacial Surgery, Kakogawa Central City Hospital, Kakogawa, Japan; 3 Department of Otolaryngology, Kakogawa Central City Hospital, Kakogawa, Japan; 4 Department of Anesthesiology, Kakogawa Central City Hospital, Kakogawa, Japan

**Keywords:** Airway management, Odontogenic severe deep neck infections, Tracheostomy, Laryngeal edema, Pharyngeal space abscess

## Abstract

In this study, we aimed to identify risk factors that predict the postoperative need for advanced or prolonged airway management in patients with severe odontogenic deep neck infections (DNIs). This retrospective case–control study included patients of both sexes aged ≥ 18 years who had undergone surgical drainage including debridement of necrotic tissues of odontogenic deep neck abscesses and necrotizing soft tissue infection under general anesthesia between April 2016 and September 2023 at a single center. The patients’ characteristics, laboratory tests, and computed tomography (CT) findings were analyzed and compared between the difficult postoperative airway group, which required prolonged intubation or tracheostomy, and the short-term intubation group. Statistical significance was set at *P* < 0.05. Sixty-four patients required surgical drainage including debridement under general anesthesia. Of them, 7 (10.9%) patients were included in the difficult postoperative airway group. In addition to increased inflammatory markers, the presence of arytenoid edema among laryngeal edema and retro- and parapharyngeal space abscesses on preoperative CT images were identified as risk factors. The presence of pharyngeal space abscesses was significantly associated with laryngeal edema, and the intubation period was longer in patients with more elements relevant to these two factors. Thus, the presence of pharyngeal space abscesses and degree of laryngeal edema on preoperative CT images can be used to predict the complexity of postoperative airway management. Our results suggest that tracheostomy is preferable for patients with retropharyngeal space abscesses, and that patients with parapharyngeal space abscesses and laryngeal edema are desirable to undergo prolonged intubation.

## Introduction

The widespread use of antibiotics has made fatal odontogenic infections rare nowadays [1]. However, they occasionally become severe and spread to deep neck spaces, called as “deep neck infections (DNIs)” [2, 3]. In fact, the most common cause of DNIs is odontogenic infection, accounting for approximately 43% of all cases [3]. Among DNIs, deep neck abscess and necrotizing soft tissue infection (NSTI) are especially severe and fatal [4, 5]. Deep neck abscesses can obstruct the airways and spread to other critical areas, such as the mediastinum, leading to life-threatening complications [4]. NSTI can cause significant tissue damage and systemic toxicity and previous studies have shown high mortality rates (approximately 42.9%) in patients infected in the maxillofacial region [5]. In the treatment of these severe DNIs, although early administration of antibiotics and surgical drainage including debridement are also needed to rescue the patient, airway management is the first priority, as swelling from the infection can obstruct breathing [6]. However, securing the airways of patients with severe odontogenic DNIs can be difficult because of the dynamic progression of the disease, which can be accompanied by severe trismus, edema of the laryngopharynx, distorted anatomy, and tissue immobility [7]. Owing to improvements in medical equipment, in addition to direct or video laryngoscopy, difficult airways can be managed using fiberoptic bronchoscopy on patients under local anesthesia [8]. However, the decisions to extubate and perform a tracheostomy are often accompanied by confusion and controversy [9]. To the best of our knowledge, this issue may arise from the lack of established standards to extubate and perform a tracheostomy in patients with severe odontogenic DNIs. Moreover, evaluation of the upper airway during tracheal intubation is often complicated by pooled saliva and pharyngeal swelling [10].

Particularly in emergency medicine, establishing standards and having a common understanding among medical professionals increase the quality of treatment, resulting in the patient rescue [11]. Before surgery for severe odontogenic DNIs, the ability to predict the degree of postoperative airway management is extremely important for appropriate airway management. In general, patients with severe odontogenic DNIs are assessed for inflammation via preoperative imaging examinations including computed tomography (CT) [12]. We mainly focused on these image findings, and oral and maxillofacial surgeons, otorhinolaryngologists, and anesthesiologists jointly retrospectively reviewed these cases retrospectively. The main objective of this study was to identify the risk factors for predicting the postoperative need for advanced or prolonged airway management in patients with severe odontogenic DNIs.

## Patients and methods

### Patients

We included patients aged ≥ 18 years, of both sexes who had undergone surgical drainage including debridement of severe odontogenic DNIs under general anesthesia at Kakogawa Central City Hospital between April 2016 and September 2023 in this study. The exclusion criteria were as follows: patients who underwent surgical drainage and debridement under local anesthesia and patients who underwent tracheostomy before surgery. The severe odontogenic DNIs included odontogenic deep neck abscesses and NSTI and their definitions were based on previous studies [13–15]. Briefly, deep neck abscesses were confirmed in the presence of abscess in the deep neck spaces on contrast-enhanced CT images. NSTI were diagnosed based on Fisher’s [16] and Mathieu’s [17] diagnostic criteria and confirmed by evidence of gas production on CT images, intraoperative findings, and histopathology.

In our hospital, the decisions to extubate and perform a tracheostomy are usually made by each anesthesiologist by comprehensively evaluating the degree of upper airway obstruction and the difficulty of reintubation including trismus, with otorhinolaryngologists, if necessary, at the end of operations and when encountering prolonged intubation. Based on medical records, all patients were divided into two groups: a difficult postoperative airway and short-term intubation groups. The difficult postoperative airway group included patients requiring prolonged postoperative intubation or tracheostomy. Prolonged intubation is typically defined as an intubation lasting longer than 48 h [18, 19]. This duration was based on the results of two previous studies that identified prolonged mechanical ventilation (longer than 48 h) as a significant risk factor for post-extubation dysphagia [20, 21]. They reported that dysphagia and aspiration occurred in approximately 50% of patients who were intubated for longer than 48 h [20, 21].

### Data collection

The following variables were retrospectively reviewed and evaluated from medical records and images: patient’s age; sex, body mass index (BMI); the presence of comorbidities such as diabetes mellitus (DM); smoking history; extent of mouth opening; inflammatory markers in blood tests such as C-reactive protein (CRP) and white blood cell (WBC) count; location of odontogenic causes, including mandibular molar; presence and degree of laryngeal edema; presence of abscess in each deep neck space; presence of gas production; and intubation period. Factors such as age, sex, BMI, comorbidities, smoking history, and trismus can influence the risk of difficult airway management [22, 23]. Blood tests for inflammatory markers are necessary to evaluate the severity of this condition [24]. According to a previous study, 96.8% of odontogenic DNIs are linked to mandibular molars [25]. The presence and degree of laryngeal edema including arytenoid edema and epiglottis swelling; presence of an abscess in each deep neck space, including para- and retropharyngeal space; and presence of gas production were investigated on plane and contrast-enhanced CT images. To evaluate laryngeal edema, laryngoscopic images were also used as a reference if taken. The imaging findings were used to evaluate the extent of inflammation. All CT images were acquired using a 64-slice CT system (Aquilion 64; Canon Medical Systems Corp, Tochigi, Japan) or 128-slice CT system (SOMATOM Definition Flash; Siemens, Munich, Germany). Data were acquired under typical head and neck CT scanning conditions (120 kV, 1–5 mm slice) with automatic exposure control. The following contrast media were used: Iomeron 300 (Eisai, Tokyo, Japan), Iopamidol 370 (Hikari Pharmaceutical, Tokyo, Japan), and Omnipaque 300 (GE Healthcare, Chicago, IL, USA). All tests, including blood tests and imaging, were performed upon admission.

**Fig. 1:**
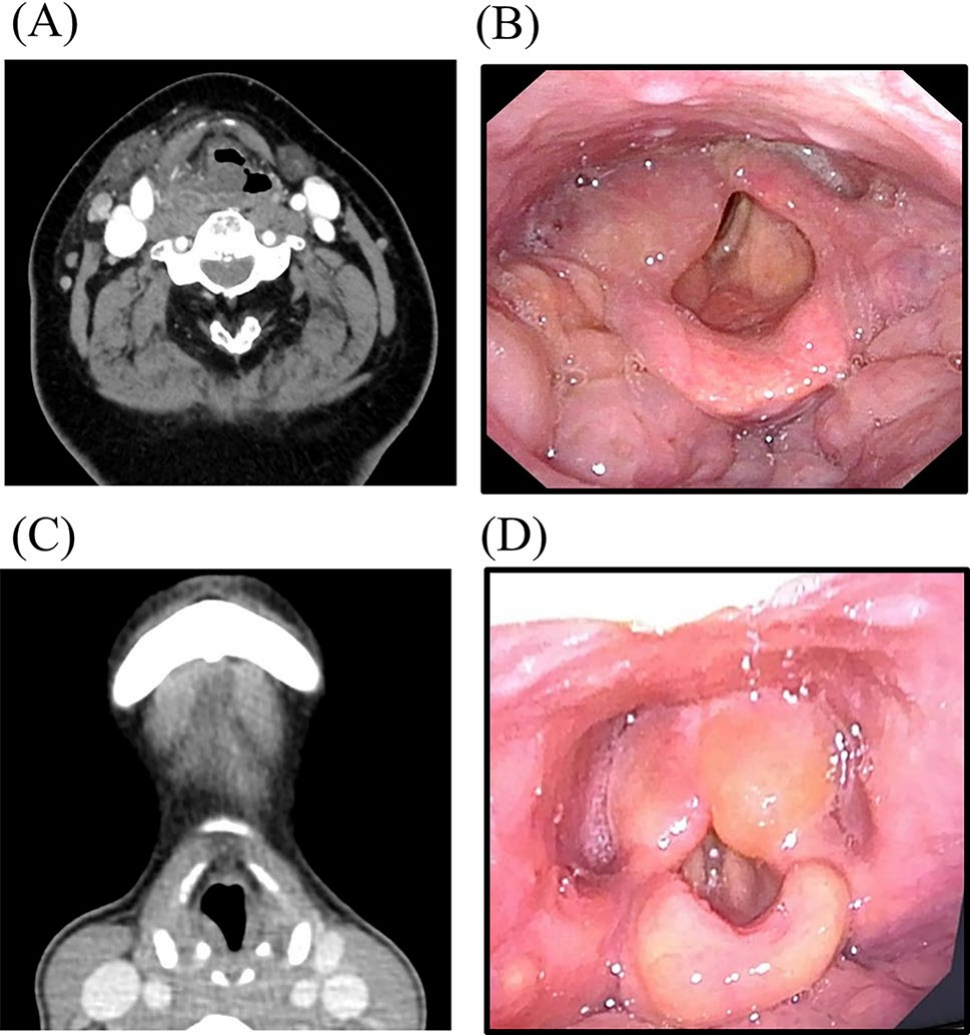
Grading of laryngeal edema (epiglottis swelling). Grade I: swelling on one side of the axial CT image (**A**) and laryngoscopy image (**B**). Grade II: swelling on both sides on axial CT image (**C**) and laryngoscopy image (**D**)

**Fig. 2:**
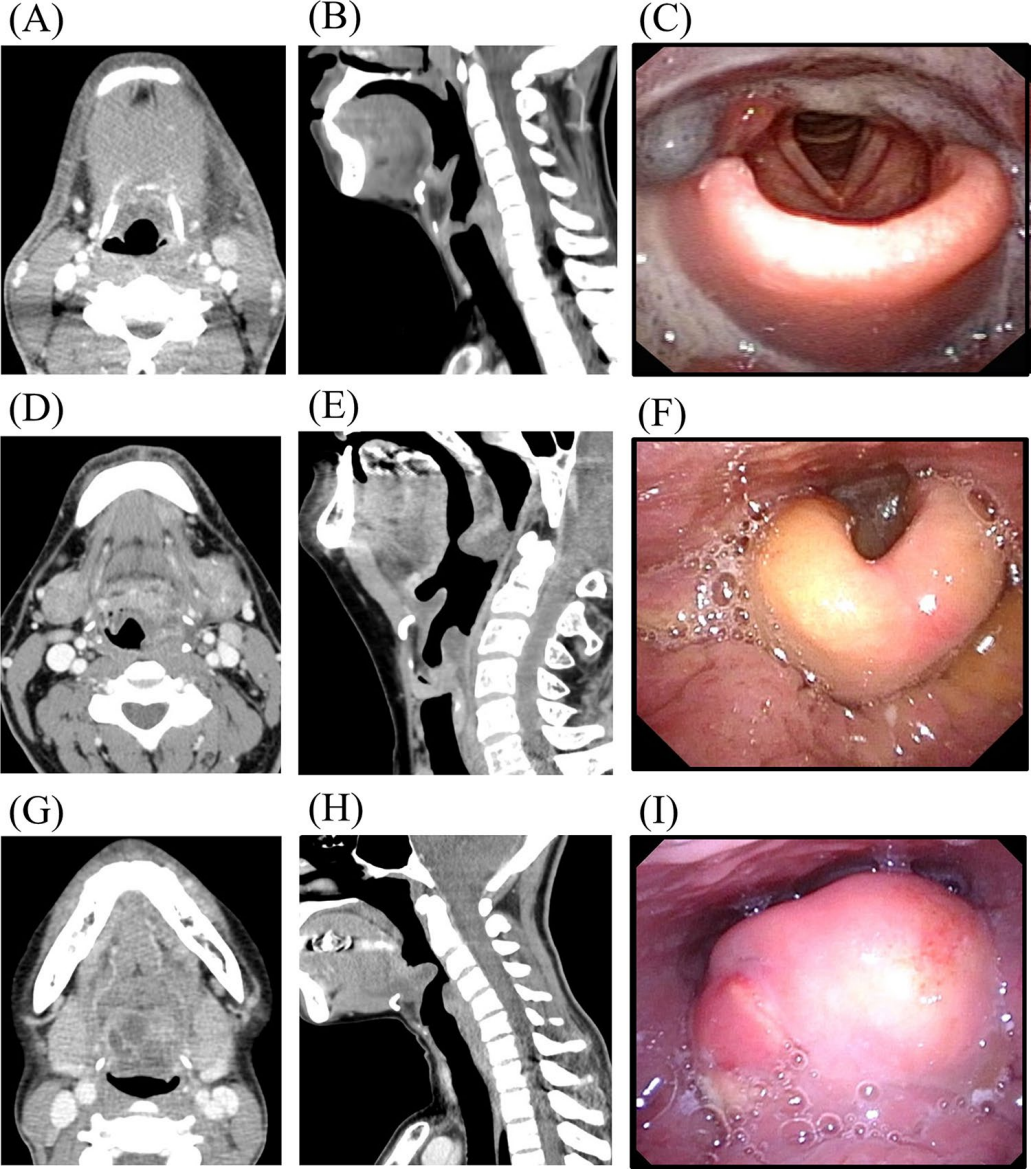
Grading of laryngeal edema (arytenoid edema). Grade I: slight swelling on axial CT image (**A**), sagittal CT image (**B**), and laryngoscopy image (**C**). Grade II: moderate swelling on axial CT image (**D**), sagittal CT image (**E**), and laryngoscopy image (**F**). Grade III: severe swelling on axial CT image (**G**), sagittal CT image (**H**), and laryngoscopy image (**I**)

To evaluate the severity of laryngeal edema, a unique grading system was created by adding CT image findings to Tanaka’s classification [26], which uses only laryngoscopy images to assess acute epiglottitis (Table 1). The classification consisted of two gradings of arytenoid edema and three gradings of epiglottic swelling. Briefly, the former consisted of the following: (1) Grade I (1 point): one side (swelling on one side) and (2) Grade II (2 points): both sides (swelling on both sides) (Fig. 1). The latter consisted of (1) Grade I (1 point): slight (swelling only on the lingual side); (2) Grade II (2 points): moderate (swelling extends to the laryngeal side and has a U-shape) and (3) Grade III (3 points): severe (epiglottis becomes spherical or heart-shaped) (Fig. 2). Total points were evaluated as “laryngeal edema score”.

**Table 1:** Comparison of difficult postoperative airway group and short-term intubation group

Variable		Difficult postoperative airway group (*n* = 7)	Short-term intubation group (*n* = 57)	*P* value
Age (years)	Median (range)	69.0 (29–80)	64.0 (19–91)	0.766^b^
Sex	Female	4 (57.1%)	28 (49.1%)	1.000^c^
BMI (kg/m^2^)	Median (range)	21.9 (17.7–28.1)	23.1 (17.5–37.9)	0.839^b^
Comorbidities	Yes	2 (28.6%)	16 (28.1%)	1.000^c^
Smoking	Smoker	1 (14.3%)	8 (14.1%)	0.980^d^
Extent of mouth opening (mm)	Median (range)	15.0 (0–25)	20.0 (5–45)	0.195^b^
CRP (mg/dL)	Median (range)	25.8 (10.4–43.4)	15.6 (1.5–30.9)	0.005*^b^
WBC (10^3^/µL)	Median (range)	20.8 (9.7–33.5)	14.1 (6.9–21.2)	0.005*^b^
Location of odontogenic cause	Mandibular molar	6 (85.7%)	42 (73.7%)	0.669^c^
Arytenoid edema	Yes	3 (42.9%)	5 (8.8%)	0.036*^c^
	Grade I	2 (28.6%)	5 (8.8%)	0.375^c^
	Grade II	1 (14.3%)	0 (1.7%)	
Epiglottis swelling	Yes	2 (28.6%)	4 (7.0%)	0.125^c^
	Grade I	0 (0.0%)	3 (5.3%)	0.082^d^
	Grade II	1 (14.3%)	1 (1.7%)	
	Grade III	1 (14.3%)	0 (0.0%)	
Laryngeal edema score (points) ^a^	Median (range)	3 (1–5)	2 (1–2)	1.000^b^
	≥ 3 (cutoff value)	2/3 (66.6%)	0/6 (0.0%)	0.083^c^
Parapharyngeal space abscess	Yes	4 (57.1%)	9 (15.8%)	0.027*^c^
Submandibular space abscess	Yes	7 (100.0%)	44 (77.2%)	0.328^c^
Sublingual space abscess	Yes	2 (28.6%)	9 (15.8%)	0.594^c^
Submental space abscess	Yes	1 (14.3%)	11 (19.3%)	1.000^c^
Pterygomandibular space abscess	Yes	4 (57.1%)	15 (26.3%)	0.182^c^
Retropharyngeal space abscess	Yes	2 (28.6%)	0 (0.0%)	0.010*^c^
Gas production	Yes	2 (28.6%)	4 (7.0%)	0.125^c^
Intubation period (days)	Median (range)	3.0 (2–24)	0.0 (0–1)	< 0.001*^b^

^a^*n* = 9: Patients with laryngeal edema. Three patients in the difficult postoperative airway group and 6 patients in the short-term group

^b^Mann–Whitney *U* test

^c^Fisher’s exact test

^d^Chi-squared test

^*^P < .05

### Statistical analysis

All statistical analyses were performed using SPSS (version 26.0; SPSS, Chicago, IL, USA) and Ekuseru-Toukei 2016 software (Social Survey Research Information Co. Ltd., Tokyo, Japan). A receiver operating characteristic (ROC) curve was used to determine cutoff values for the laryngeal edema score. The area under the ROC curve (AUC) was used to measure discrimination accuracy. The association of each variable with a difficult postoperative airway was analyzed using the non-parametric Mann–Whitney *U* test for ordinal variables, and Fisher’s exact test or the Chi-squared test was used for categorical variables. *P* < 0.05 was considered statistically significant.

## Results

Table 1 shows the comparison between the difficult postoperative airway and short-term intubation groups. Sixty-four patients required surgical drainage including debridement under general anesthesia. Seven (10.9%) patients were included in the difficult postoperative airway group. For the laryngeal edema score, the cutoff value was determined in 9 patients with laryngeal edema using the corresponding ROC curve. A laryngeal edema score ≥ 3 points had a sensitivity of 66.7%, specificity of 100.0%, and AUC of 0.722 (95% confidence interval 0.173 to 1.271). In addition to increased levels of inflammatory markers in blood tests, the presence of arytenoid edema among laryngeal edema and pharyngeal space abscesses (parapharyngeal and retropharyngeal space) on CT images showed significant differences between the two groups (Table 1).

Table 2 shows the differences in preoperative CT findings between the groups with and without pharyngeal space abscesses. The presence of pharyngeal space abscesses was significantly associated with the presence of arytenoid edema, laryngeal edema, and gas production on preoperative CT images.

**Table 2:** Differences of preoperative CT findings in groups with or without pharyngeal space abscess

Variable		Pharyngeal space abscess group (*n* = 13)	Non-pharyngeal space abscess group (*n* = 51)	*P* value
Arytenoid edema	Yes	5 (38.5%)	3 (5.9%)	0.007*^c^
	Grade I	4 (30.8%)	1 (2.0%)	0.464^c^
	Grade II	1 (7.7%)	2 (3.9%)	
Epiglottis swelling	Yes	2 (15.4%)	4 (7.8%)	0.593^c^
	Grade I	1 (7.7%)	2 (3.9%)	0.223^d^
	Grade II	0 (0.0%)	2 (3.9%)	
	Grade III	1 (7.7%)	0 (0.0%)	
Laryngeal edema score (points)^a^	Median (range)	1 (1–5)	2 (2–3)	0.400^e^
	Yes	5 (38.5%)	4 (7.8%)	0.013*^c^
Gas production	Yes	4 (30.8%)	2 (3.9%)	0.013* ^c^
Difficult postoperative airway group	Yes	4 (30.8%)	3 (5.9%)	0.027*^c^
Intubation period (days) ^b^	Median (range)	4.0 (1–24)	2.0 (1–3)	0.143^e^

^a^*n* = 9: Patients with laryngeal edema. Five patients were in the pharyngeal space abscess group, and 4 patients were in the non-pharyngeal space abscess group

^b^*n* = 10: Patients with postoperative intubation or tracheostomy

^c^Fisher’s exact test

^d^Chi-squared test

^e^Mann–Whitney *U* test

^*^P < .05

Clinical and imaging findings of patients in the difficult postoperative airway group are shown in Table 3. All patients had severe trismus (< 20 mm). One of the seven patients (14.3%) underwent tracheostomy, whereas the others were managed with intubation. One patient was managed with intubation for more than 7 days. After surgery, all 7 patients were admitted to the intensive care unit and underwent multimodal therapy including antibiotic therapy. Five patients other than the two patients with DM were also treated with hydrocortisone as an adjuvant treatment for antibiotic therapy. The intubation period was longer in cases with more factors related to laryngeal edema and pharyngeal space abscess. The most common reason why anesthesiologists decided not to extubate postoperatively was laryngeal edema, followed by pharyngeal edema and severe trismus (data not shown).

**Table 3:** Clinical and image findings of patients in difficult postoperative airway group

Patient no	Age	Comorbidities	Extent of mouth opening (mm)	CRP	Arytenoid edema	Epiglottis swelling	Laryngeal edema score	Parapharyngeal space abscess	Retropharyngeal space abscess	Gas production	Intubation period (days)
1	41	–	10	10.4	–	–	0	–	–	–	2
2	29	–	0	21.4	–	–	0	** + **	–	** + **	2
3	72	DM	10	36.5	–	–	0	–	–	–	3
4	39	–	15	17.7	+ (I)	+ (II)	3	–	–	–	3
5	80	–	15	28.9	–	–	0	** + **	–	–	4
6	69	–	25	25.8	+ (II)	+ (III)	5	** + **	** + **	–	8
7	75	DM	20	43.4	+ (I)	–	1	** + **	** + **	** + **	24 (tracheostomy)

*DM* diabetes mellitus

## Discussion

In this study, we identified risk factors that predict the need for advanced or prolonged airway management after surgical drainage, including debridement of severe odontogenic DNIs. Seven (10.9%) patients required prolonged intubation or tracheostomy. In addition to increased levels of inflammatory markers, the presence of arytenoid edema among laryngeal edema and pharyngeal space abscesses were also identified as risk factors. The presence of a pharyngeal space abscess was significantly associated with laryngeal edema, and the intubation period was longer in patients with more characteristics related to these two factors.

First, we focused on the range of abscess formation. The scoring system proposed by Flynn et al. [27, 28] is a well-known and useful classification system for measuring the severity of odontogenic infections. This system consists of three categories and assigns a severity score in each deep neck space based on the severity of the odontogenic infection, as follows: (1) low-risk spaces (score 1): vestibular, subperiosteal, infraorbital, and buccal space; (2) medium-risk spaces (score 2): submandibular, submental, sublingual, pterygomandibular, sub-masseteric, superficial temporal, and deep temporal; (3) high-risk spaces (score 3): lateral pharyngeal, retropharyngeal, pre-tracheal, danger space, mediastinum, and intracranial space [27, 28]. The total scores offer valuable insights into the extent of infection based on the risk posed to airway function. Neal et al. investigated 115 patients with DNIs caused by severe odontogenic infections and found that, based on the scoring system by Flynn et al., patients with total scores ≥ 5 were associated with a higher prevalence of difficult intubations compared to patients with total scores < 5 [29]. Nagaura et al. investigated 113 patients with severe odontogenic infections and found that the presence of inflammation that had spread to high-risk spaces of the Flynn et al. scoring system, especially the parapharyngeal space, on preoperative CT images was an important indicator of the difficulty posed by intubation [30]. Chen et al. investigated 403 patients with DNIs and reported that the parapharyngeal and retropharyngeal spaces were most commonly involved in patients with DNIs that required tracheostomy [31]. Yuan et al. investigated 81 patients with DNIs caused by non-odontogenic infections and reported that the involvement of the infrahyoid neck space was a high-risk factor for the management of DNIs [32]. In the present study, pharyngeal space abscesses were significantly associated with postoperative airway difficulty. Two patients with abscess formation in the retropharyngeal space located in the infrahyoid were included in the difficult postoperative airway group. These results suggest that retropharyngeal space abscesses in the pharyngeal space are particularly important risk factors for difficult postoperative airway management.

We also focused on the degree of laryngeal edema. To the best of our knowledge, no previous study has investigated the degree of laryngeal edema in patients with severe odontogenic DNIs. In patients with acute epiglottitis, the degree of arytenoid edema and epiglottis swelling is typically evaluated [26, 33]. In a previous study, Katori et al. investigated 96 Japanese patients with acute epiglottitis and identified the diagnosis using a laryngoscope as a useful technique; they found that patients with severe epiglottis swelling accompanied by arytenoid edema required aggressive airway management [33]. In another study, Tanaka et al. investigated 285 Japanese patients with acute epiglottitis and reported that their unique classification was beneficial in determining the indications for airway management [26]. All patients who complained of severe dyspnea or underwent tracheotomy or cricothyroidotomy scored over four points, while none of the patients with less than three points disease required aggressive airway management [26]. In this present study, all the patients in the short-term intubation group scored less than two points. In contrast, two patients in the difficult postoperative airway group scored more than three points. One patient had one point, but suffered from a retropharyngeal space abscess. The lower cutoff points compared to previous studies (3 vs. 4 points) may be due to the differences in the distances to the larynx between odontogenic infections and acute epiglottitis and consideration for prolonged intubation. Our results suggest that a laryngeal edema score ≥ 3 is an important risk factor not only for difficult tracheal intubation, but also for prolonged intubation.

We also examined steroid use. Steroids are a commonly used to treat acute infections including DNIs and laryngeal edema [34]. Some studies have reported the effect of steroids as adjuvant treatment for antibiotic therapy in DNIs [35–38]. Tansey et al. investigated 153 patients with DNIs who were treated with dexamethasone and without dexamethasone in addition to antibiotic therapy and reported that the dexamethasone group underwent more surgical drainage (36% vs. 53%, *P* = 0.043) and had shorter mean length of stay (2.9 days vs. 3.8 days, *P* = 0.09) [36]. Villanueva-Fernandez et al. investigated 30 children with pharyngeal abscesses who were treated with intravenous antibiotics and corticosteroids and reported that the length of hospital stay was reduced with no complications [37]. Konishi et al. investigated 1,882 patients with pharyngeal abscesses and reported that steroid use may provide pain relief from sore throat without serious adverse events and reduce surgical drainage [38]. Their study included patients with DM, but the use of steroids should be taken into account because it causes hyperglycemia and may precipitate diabetic ketoacidosis (DKA), which is a life-threatening condition linked to 16% of deaths related to DM [39]. In the present study, 2 patients with DM were not treated with hydrocortisone. DKA is a life-threatening complication of DM and typically presents as elevated blood glucose (250–600 mg/dL), bicarbonate < 18 mmol/L, elevated anion gap (> 10 mEq/L), moderate to large urine ketones, and elevated serum beta-hydroxybutyrate with acidosis [39]. Huang et al. investigated 185 patients with DNIs and reported that, among them, patients with DM had significant abscess formation (89.3% vs. 71.6%, *P* = 0.014), underwent surgical drainage (86.0% vs. 65.2%, *P* = 0.014), and more frequently underwent tracheostomy or intubation (19.6% vs. 6.2%, *P* = 0.012) than without DM [40]. Therefore, because patients with DM are susceptible to DNIs that tend to become more severe, the management of DNIs is especially complicated. To the best of our knowledge, there are no guideline for the treatment of odontogenic DNIs in patients with DM, especially regarding DKA. However, a previous study identified steroid use as a risk factor for developing DKA [41]. Mondal et al. reported a significant association between the development of DKA in patients with BMI < 25.56 kg/m^2^, hemoglobin A1c > 8.35 mg/dL, and IL-6 levels > 50.95 pg/mL [41]. These results suggest that steroids may be useful in patients with DNIs as an adjuvant treatment for antibiotic therapy but in patients with DM, careful attention is needed to monitor and treat elevated glucose levels and the risks of DKA development. If the two patients with DM in this study were treated with hydrocortisone, the intubation period may have decreased. Such cases should be investigated in the future.

Furthermore, the decision of whether to perform a tracheotomy or prolonged intubation is debatable. In general, the advantages of tracheotomy include airway security, better patient comfort, less need for sedation and mechanical ventilation, and earlier transfer to a noncritical care unit, resulting in lower intensive care costs and lower overall hospitalization costs [42, 43]. However, it is a surgical procedure with potential postoperative complications such as scarring, bleeding, pneumothorax, and tracheal stenosis [44]. The advantages of tracheal intubation include the fact that it is the fastest method of airway control and is a nonsurgical procedure. Disadvantages include complexity in cases of upper airway edema, patient discomfort, and the need for sedation and mechanical ventilation [44]. This study focused on patients whose airways were secured under general anesthesia. A recent systematic review on the timing of tracheostomy in patients with prolonged endotracheal intubation reported that tracheostomy within 7 days of intubation is desirable due to lower rates of mortality and hospital-acquired pneumonia [44]. In the present study, one patient with ≥ 7 days of intubation had Grade II of arytenoid edema, Grade III of epiglottis swelling (i.e., Laryngeal edema score: five points), and posterior pharyngeal abscesses on preoperative CT images. Another patient who underwent tracheostomy had Grade I arytenoid edema (i.e., Laryngeal edema score: one point) and retropharyngeal abscess. Our results suggest that the presence of retropharyngeal space abscesses is a particularly important risk factor indicative of the necessity for tracheostomy in patients with severe odontogenic DNIs, because of its ability to predict a longer duration of intubation.

**Fig. 3:**
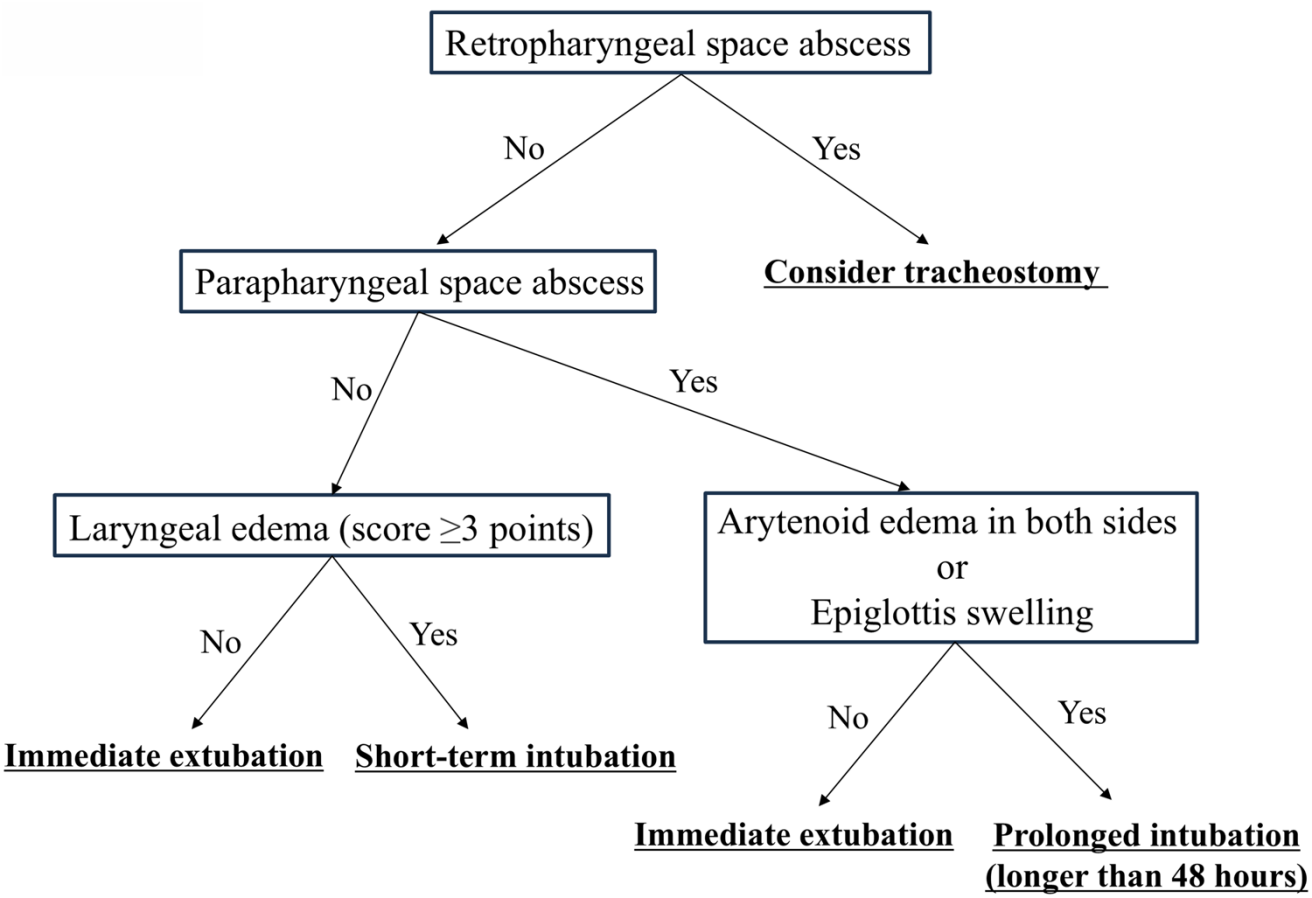
Proposed airway management strategy for severe odontogenic DNIs

Finally, we propose a risk-stratification method for airway management in patients with severe odontogenic DNIs based on our results (Fig. 3). First, patients were evaluated based on the presence of retropharyngeal and parapharyngeal space abscesses on contrast-enhanced CT images. Next, they were evaluated for the presence and degree of laryngeal edema using CT or laryngoscopy, if needed. Depending on the resulting severity, patients are classified into four grades: “consider tracheostomy,” “prolonged intubation (longer than 48 h),” “short-term intubation,” and “immediate extubation”. Although most patients were successfully matched to a specific grade, some patients were not perfectly matched. Four of seven patients in the difficult postoperative airway group (patients no. 1, 2, 3, and 5 in Table 3) were matched with either “short-term intubation” or “immediate extubation”. For instance, patient no. 2, who had parapharyngeal abscesses but not laryngeal edema, was managed via intubation for 2 days but was matched to “short-term intubation” in the tree diagram. The patient was unable to open their mouth at all preoperatively and had an NSTI with gas production on preoperative CT. The anesthesiologist determined that extubation should not be performed at the end of the operations because of airway deviation caused by the parapharyngeal space abscesses and severe trismus. Although severe trismus is known to be a predictor of difficult reintubation [45], the other three patients were unable to open their mouths beyond 15 mm. Although our proposed risk-stratification method for airway management can identify patients who require prolonged intubation or tracheostomy, it should be applied on a case-by-case basis to patients with other airway risks, including severe trismus. In the present study, increased levels of inflammatory markers were identified as risk factors. Although inflammatory markers are useful for assessing the severity of odontogenic DNIs, they assess systemic inflammatory response not just in the airways [24]. Therefore, we conducted tree diagram that focused on the imaging findings to evaluate the airway condition.

This is the first study to investigate the degree of laryngeal edema as a predictive factor for postoperative airway management in patients with severe odontogenic DNIs. To the best of our knowledge, there are no established standards to extubate and perform a tracheostomy in those patients. Therefore, oral and maxillofacial surgeons, otorhinolaryngologists, and anesthesiologists jointly retrospectively reviewed cases (valuable in itself) and proposed a risk-stratification method for airway management in patients with severe odontogenic DNIs. We consider this risk-stratification method valuable for future emergency medicine and plan to work with this standard as a common understanding in our hospital. This may be useful for not only medical professionals and but the patients and their family for informed consent before surgery. However, this study had several limitations. First, as a retrospective study, there is a possibility of unknown confounding factors including vital signs. Second, the population and number of outcomes evaluated in this study were small, which may have introduced bias through data selection and analyses. However, severe odontogenic DNIs requiring tracheostomy or prolonged intubation are rare. Finally, the decision to extubate may differ among anesthesiologists. However, a single anesthesiologist cannot handle all operations alone, surgery for severe odontogenic DNIs is an emergency procedure and not elective. All the anesthesiologists who participated in this study had sufficient experience. In the future, we plan to conduct a multicenter study with a larger sample size to investigate whether our results can be generalized. However, variations in clinical practice across institutions should also be acknowledged. In addition, we plan to conduct a similar study on severe DNIs caused by factors other than odontogenesis, including the tonsil.

## Conclusion

We found that the presence of pharyngeal space abscesses and the degree of laryngeal edema observed on preoperative CT images could be used to predict the complexity of postoperative airway management. Our results suggest that tracheostomy is preferable for patients with retropharyngeal space abscesses, and that patients with parapharyngeal space abscesses and laryngeal edema are desirable to undergo prolonged intubation.

## Data Availability

The datasets can be obtained from the corresponding author upon reasonable request.
